# Lung proliferative lesion‐promoting effects of left pulmonary ligation in A/J female mice

**DOI:** 10.1111/pin.12915

**Published:** 2020-02-21

**Authors:** Masanao Yokohira, Nozomi Hashimoto, Keiko Yamakawa, Yuko Nakano‐Narusawa, Yoko Matsuda, Katsumi Imaida

**Affiliations:** ^1^ Oncology Pathology, Department of Pathology and Host‐Defense, Faculty of Medicine Kagawa University Kagawa Japan

**Keywords:** A/J mouse, IGF‐1, ligation, lung proliferative lesion, NNK

## Abstract

This present study was conducted in an attempt to examine proliferative lesion‐promoting effect in the lung by compensatory lung growth after left pulmonary ligation. To examine a strong proliferative lesion‐promoting effect in the lung, the effects of left pulmonary ligation on lung proliferative lesions induced by 4‐(methylnitrosamino)‐1‐(3‐pyridyl)‐1‐butanone (NNK) were examined for 12 weeks. The number of proliferative lesions induced by NNK in the right lung after left pulmonary ligation increased significantly after 12 weeks, indicated by an increase in the weight of the right lung. In addition, several messenger RNA (mRNA) markers, including insulin growth factor 1, were highly expressed in the right lung on the seventh day after left ligation. These experiments demonstrated the clear proliferative lesion‐promoting effects of pulmonary ligation on the induction of the expression of mRNAs related to the cell cycle, cell division and mitosis. However, the proliferative lesion‐promoting effects were not strong enough to allow a shortened experimental period for the establishment of the lung bioassay model. The results also indicated the necessity to pay attention to the possibility of a recurrence of lung cancer in the residual lung after resection in humans.

**Abbreviations**
ABC: avidin‐biotin complex
Cdc25c: cell division cycle 25C
Ckap2: cytoskeleton‐associated protein 2
GH: growth hormones
GST‐P: glutathione S‐transferase placental form
IGF: insulin growth factor 1
Mast1: microtubule‐associated serine/threonine kinase‐like
NNK: 4‐(methylnitrosamino)‐1‐(3‐pyridyl)‐1‐butanone

## INTRODUCTION

Lung cancer is the most common cause of cancer‐related deaths in the world, with cigarette smoking regarded as its major cause.^1^ However, the risk of lung cancer development remains elevated even when one quits smoking, as inhalation of second‐hand smoke continues to be a problem.^2^, ^3^ To identify potential chemopreventive agents for lung cancer, the creation of bioassay models of the lung is important. We have been studying with a focus on establishing a short‐term lung bioassay model for the identification of chemopreventive agents acting during the initiation phase of lung carcinogenesis.^4^ Previous studies examined the shortest period for assessing the effects of test agents on 4‐(methylnitrosamino)‐1‐(3‐pyridyl)‐1‐butanone (NNK)‐induced lung tumor development, and 12 weeks was shown to be effective for the detection of lung cancer chemoprevention.^4^ NNK is a tobacco‐specific N‐nitrosamine that conceivably plays an important role in tobacco‐related lung cancer in humans, given its strong ability to induce lung tumorigenesis in rodents.^1^, ^5^, ^6^, ^7^, ^8^, ^9^

Liver regeneration after partial hepatectomy is one of the most commonly studied models of cell, organ and tissue regeneration.^10^ The medium‐term bioassay of the liver is very useful for the detection of hepatocarcinogenicity.^11^ The 8‐week protocol consists of two stages; male F344 rats are given a single i.p. injection of diethylnitrosamine (200 mg/kg) for the induction of liver carcinogenesis, followed by a 6‐week test of chemical treatment, 2 weeks after the injection.^12^ Test chemicals are usually administered through food or drinking water; in the second week of the chemical treatment test, all rats are subjected to two‐thirds partial hepatectomy to induce regenerative cell replication.^13^, ^14^ The end‐point markers are GST placental form (GST‐P)‐positive hepatic foci, the numbers and sizes of which are expressed as values per unit liver section.^12^, ^15^ This bioassay uses compensatory regeneration by partial hepatectomy to induce tumor‐promoting effects and eventually reduce the experimental period.

This present study was conducted in an attempt to examine proliferative lesion‐promoting effect in the lung by compensatory lung growth after left pulmonary ligation, as in the medium‐term liver bioassay.^4^, ^16^ Compensatory lung growth after pneumonectomy has been reported to occur,^17^, ^18^ and in the present experiment, pulmonary ligation was substituted for pneumonectomy. If a strong proliferative lesion‐promoting effect was found in the lung by left pulmonary ligation, it could shorten the experimental period from the 12 weeks proposed by previous studies in a lung bioassay model.^4^ The effect of left pulmonary ligation in exerting a promoting effect in NNK‐induced lung proliferative lesions in 12 weeks was examined (experiment 1). In addition, to investigate the role of mechanical signals in the induction of gene expression, messenger RNA (mRNA) expression in the right lung was also examined using microarray analysis on the seventh day after left pulmonary ligation (experiment 2).

## MATERIALS AND METHODS

### Chemicals

The NNK (Cas No., 64091‐91‐4; purity, 98%) was purchased from Toronto Research Chemicals (Toronto, Canada) and was suspended in saline (Otsuka isotonic sodium chloride solution; Otsuka Pharmaceutical Factory, Tokushima, Japan).

### Animals

Female A/J mice (5 weeks old) were purchased from Japan SLC (Shizuoka, Japan) and maintained in the Division of Animal Experiments, Life Science Research Center, Kagawa University, according to the Institutional Regulations for Animal Experiments. The regulations observed included the best considerations of animal welfare and good animal handling practices, contributing to the replacement, refinement, and reduction of animal testing (3Rs). The protocols of the experiments were approved by the Animal Care and Use Committee for Kagawa University. The animals were housed in polycarbonate cages, with re‐used paper chips (EchoChip, CL‐4163; CLEA Japan, Tokyo, Japan) for bedding. They were given free access to drinking water and a basal diet, Oriental MF (Oriental Yeast Co., Tokyo, Japan), under controlled conditions of humidity (60 ± 10%), lighting (12‐hour light/dark cycle), and temperature (24 ± 2°C). The experiments were started after a 2‐week acclimatization period.

### Experimental design

#### Experiment 1

**Figure 1:**
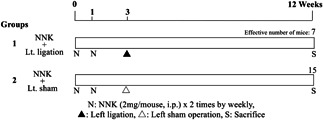
Design of experiment 1 for testing the effects of left pulmonary ligation to the lung tumor induced by 4‐(methylnitrosamino)‐1‐(3‐pyridyl)‐1‐butanone (NNK).

**Figure 2:**
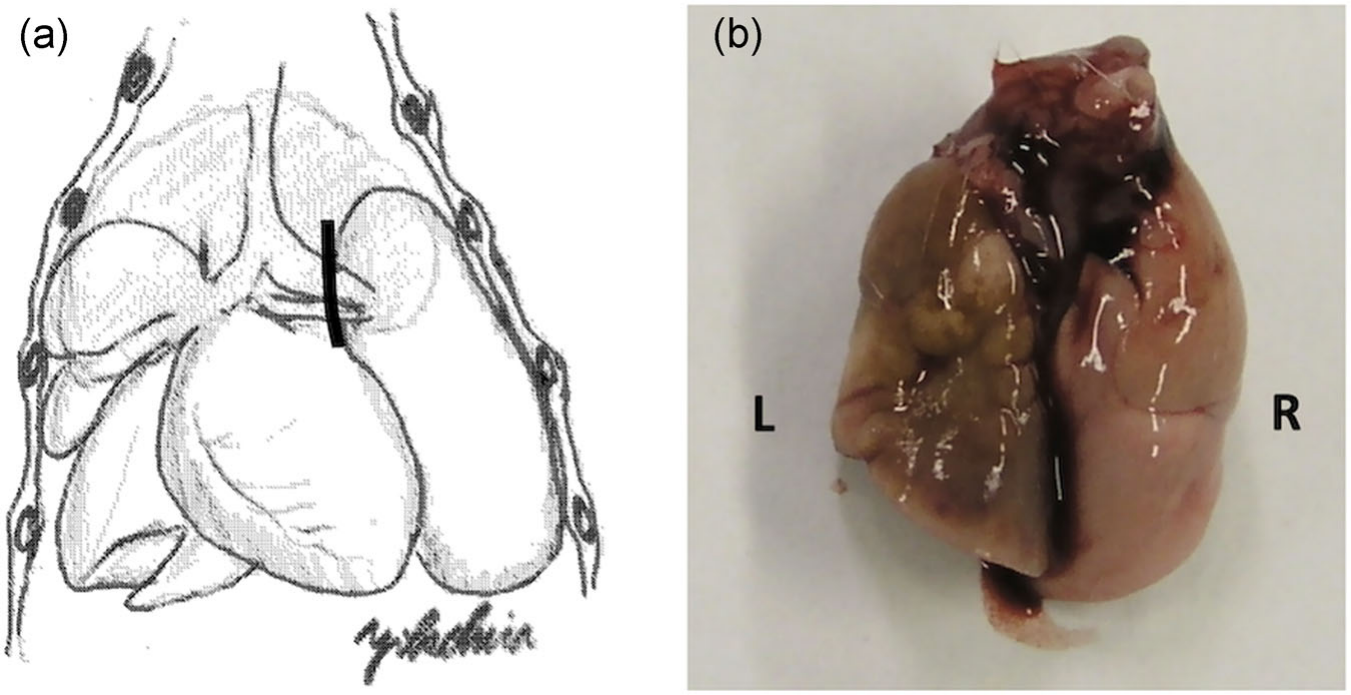
(**a**) The schema of the operation. Solid line indicates the location of clipping. (**b**) Macroscopic finding on week 12 in experiment 1. The left lung (L) appears completely collapsed.

A total of 66 7‐week‐old mice were divided into two groups of 51 (group 1) and 15 (group 2) mice, and were pretreated with NNK (2 mg/0.1 mL saline; mouse i.p.)^4^ at weeks 0 and 1 (Figure 1). At week 3, the 51 mice of group 1 underwent left pulmonary ligation. The left lung, but not the right, was chosen for ligation because the left lungs of mice consist of one lobe and a prominent pulmonary hilum. The procedure was as follows. Each mouse was given an i.p. injection of 0.2 mL pentobarbital sodium (Nembutal, Dainippon Sumitomo Pharma Co., Osaka, Japan) diluted 10 times (0.06–0.1 mL/10 g body weight). Under deep anesthesia, a skin incision (approximately 7 mm long) was made in the left axilla. After confirmation of the location of the thoracic wall, thoracotomy was completed with an incision (approximately 5 mm long) between the ribs^16^ (Figure 2a). The left lung was observed directly through this incised opening to confirm atelectasis. The left pulmonary hilum was tied up using a clipping device, Hemoclip manual‐load appliers (No. 17‐085‐01, Mizuho Corp., Tokyo, Japan) and Hemoclip‐cartridge (No. 17‐088‐01; Mizuho Corp.). Finally, the opened chest wall was clipped to close the thorax. The experiment was terminated after 12 weeks and all mice of each group were killed under deep anesthesia.

#### Experiment 2

**Figure 3:**
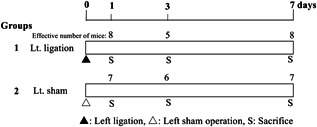
Design of experiment 2 for the microarray analysis of the right lung with left pulmonary ligation.

A total of 83 mice at 7 weeks of age were divided into two groups of 63 (group 1) and 20 (group 2) mice (Figure 3). At week 0, the 63 mice of group 1 underwent left thoracotomy and left pulmonary ligation (the procedure was almost the same as in experiment 1); group 2 mice underwent a sham operation, left thoracotomy, and clipping of the opened chest wall just after macroscopic confirmation of the lung. The experiment was terminated after 1, 3 and 7 days and the mice were killed under deep anesthesia on each day.

### Tissue preparation in experiment 1

At autopsy, the lungs were excised, weighed and then infused with 10% neutrally buffered formalin and carefully inspected grossly. After fixation, all macroscopically detected lung nodules were counted under a stereomicroscope. The seven mice with completely collapsed left lungs were counted to determine the effective number with left ligation in group 1 (Figure 2b). Due to the surgical deaths and few mice with completely collapsed left lung, the effective number was 7. The lungs were sliced routinely and processed with paraffin embedding for histopathological examination of hematoxylin and eosin‐stained sections. Lung hyperplasia and adenoma were diagnosed according to the criteria of the International Classification of Rodent Tumors: The Mouse.^19^

### Microarray analysis in experiment 2

At autopsy, the lungs were excised, weighed and then frozen immediately for microarray analysis. The mice with completely collapsed left lungs were counted to determine the effective number with left ligation in group 2. Frozen right lung samples from three mice per group on day 7 were used for microarray analysis. Six microarray datasets were obtained from single‐color hybridization of murine RNAs on Agilent Whole Mouse Genome Oligo Microarrays and Agilent Technologies Japan (Tokyo, Japan), after T7 RNA amplification. The samples were derived from animal tissues in three control and three treated conditions. The main focus of the analysis was to identify differential expression between the treated and control conditions, which was achieved by a comparison of the expression profiles of the samples in these conditions. The hybridization procedure was performed according to the Agilent 60‐mer oligo microarray processing protocol, using the Agilent Gene Expression Hybridization Kit (Agilent Technologies Japan). Briefly, 1.65 μg Cy3‐labeled fragmented cRNA in hybridization buffer was hybridized overnight (17 h, 65°C) to Agilent Whole Mouse Genome Oligo Microarrays 4x44K, using a hybridization chamber and oven. Finally, the microarrays were washed once with the Agilent Gene Expression Wash Buffer 1 for 1 min at room temperature, followed by a second wash with preheated Agilent Gene Expression Wash Buffer 2 (37°C) for 1 min. The last washing step was performed with acetonitrile. During the analysis, the dataset was processed as follows: preprocessing of the data, global correlation analysis of the unfiltered dataset, and statistical and non‐statistical analyses to identify expression differences between the treated and control conditions. The data were analyzed using the web‐software, DAVID Bioinformatics Resources 6.8 (https://david.ncifcrf.gov), which performed functional annotation clustering.

### Immunohistochemistry in experiment 1

The lungs and livers in experiment 1 were immunostained to measure insulin growth factor 1 (IGF‐1) levels using the avidin‐biotin complex (ABC) method. All staining processes from deparaffinization to counterstaining with hematoxylin were performed automatically using the Ventana Discovery XT system (Ventana Medical Systems, Tucson, AZ, USA). Antigen retrieval was performed, using the ‘extended mode’ with RiboCC buffer (Ventana Medical Systems) and the IGF‐1 rabbit polyclonal antibody for mice (bs‐0014R; Bioss Antibodies, Woburn, MA, USA), were used at a 1:25 dilution ratio with a reaction time of 60 min.

### Statistical analysis

The weights of the body and lung, as well as multiplicity of lung proliferative lesions, were analyzed using the Student's *t*‐test. The incidence of lung proliferative lesions was analyzed using the Fisher's exact probability test and multiplicity of data using the Student's *t*‐test. *P* values less than 0.05 were considered significant.

## RESULTS

### Experiment 1 for lung proliferative lesion analysis after 12 weeks

Due to bleeding from the operation, a total of 22 mice in group 1 were dead before coming out from the anesthetic. Though 29 mice were killed at the termination of the experiment after 12 weeks, only seven mice with completely collapsed left lungs were counted to determine the effective number with left ligation in group 1. The left ligated lung was brown‐green colored and showed necrotic changes macroscopically (Figure 2b). Absolute and relative weights of the right lung in group 1 with left pulmonary ligation were 0.12 ± 0.02 g and 0.48 ± 0.06 g, respectively. They were significantly higher than those (0.09 ± 0.02 g and 0.38 ± 0.03 g) of the right lung in group 2, which underwent sham operations. Whitish lung nodules were macroscopically observed in both groups. Group 1 mice with ligated left lungs had significantly more nodules in the right lung (5.4 ± 3.0) than those of group 2, (2.5 ± 2.4) (Table 1). Hyperplasia of the right lung in mice with ligated left lungs from group 1 (2.6 ± 1.7) was significantly increased compared with that in group 2 (1.2 ± 1.2). In contrast, adenoma of the right lung appeared to be more frequent in group 1 (0.4 ± 0.8) than in group 2 (0.3 ± 0.5), although the difference was not significant.

**Table 1:** Macroscopical and histopathological lesions in the lung (experiment 1)

			1	2
		Groups	NNK + Left ligation	NNK + Left sham
		No.	7	15
Macroscopic lung nodules				
		Right	5.4±3.0*	2.5±2.4
		Left	0.0±0.0	1.3±2.3
		Bilateral	5.4±3.0	3.9±4.5
Histopathological lung lesions				
	Hyperplasia			
		Right	2.6±1.7*	1.2±1.2
		Left	0.0±0.0	0.4±0.6
		Bilateral	2.6±1.7	1.6±1.5
	Adenoma			
		Right	0.4±0.8	0.3±0.5
		Left	0.0±0.0	0.1±0.4
		Bilateral	0.4±0.8	0.4±0.5
	Hyperplasia + adenoma			
		Right	3.3±1.9*	1.5±1.4
		Left	0.0±0.0	0.5±0.6
		Bilateral	3.0±1.9	2.0±1.4

* *P* < 0.05 vs. NNK with sham operation group.

Abbreviation: NNK4‐(methylnitrosamino)‐1‐(3‐pyridyl)‐1‐butanone.

### Experiment 2 for microarray analysis after short period

**Figure 4:**
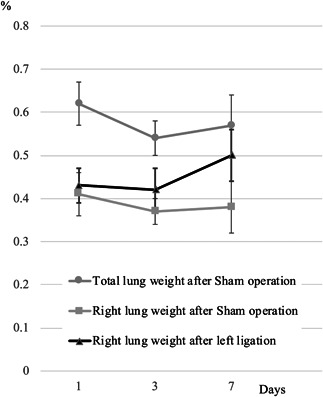
Relative lung weights in experiment 2. Right lung weights of the group with left ligation increased with time and were similar to the bilateral lung weights of the group with sham operation on day 7.

**Figure 5:**
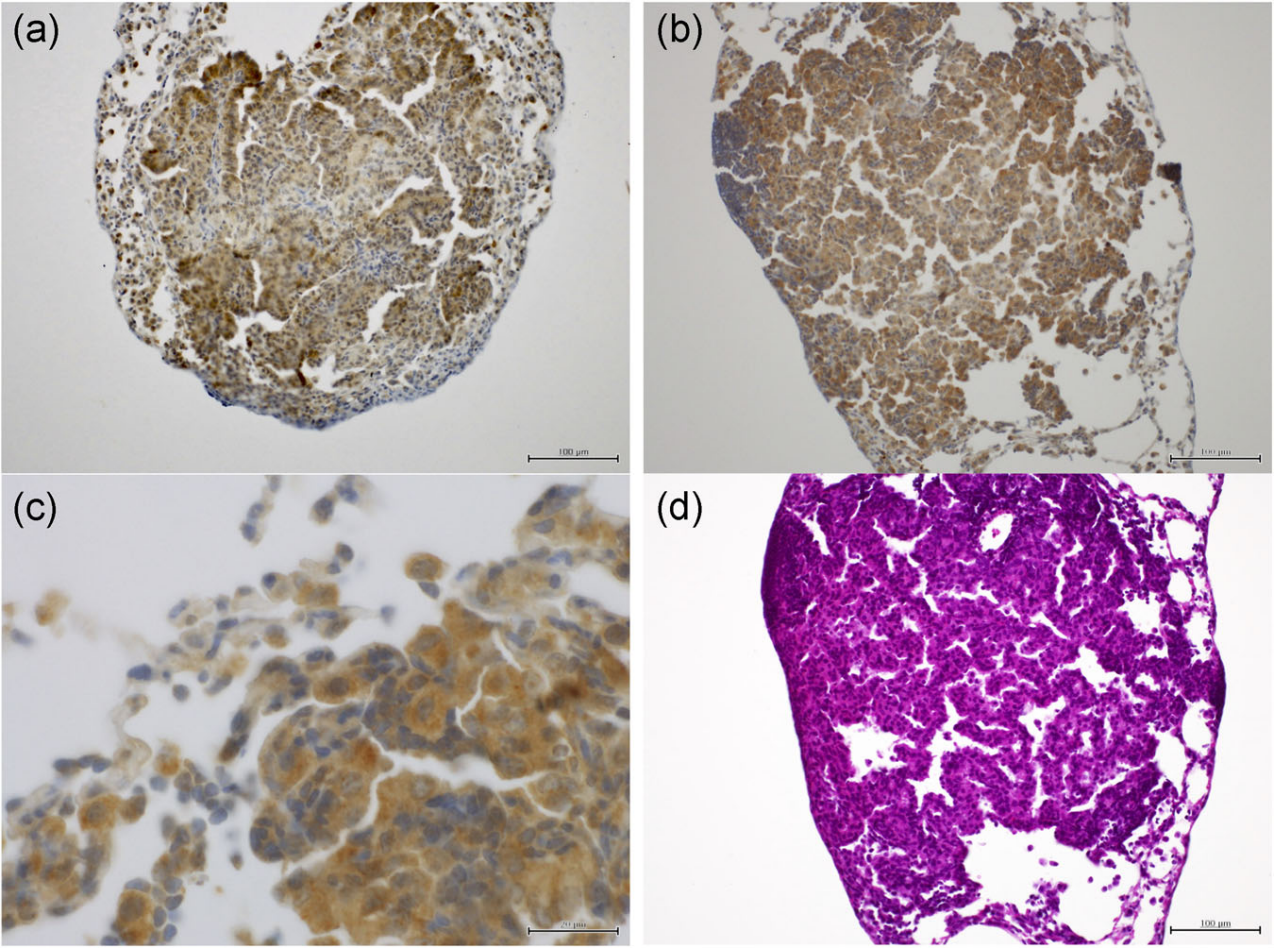
Immunohistochemistry for IGF‐1 (experiment 1). (**a**) Insulin growth factor 1 (IGF‐1) expression in the lung adenoma in the right lung (left sham operation). Scale bar: 100 μm; (**b**) IGF‐1 expression in the lung adenoma in the right lung (left ligation). Scale bar: 100 μm; (**c**) High magnification of IGF‐1 expression in the lung adenoma in the right lung (left ligation). Scale bar: 20 μm; (**d**) H.E. section of the lung adenoma in the right lung (left ligation), Scale bar: 100 μm. The strong expression of IGF‐1 was observed in the lung hyperplasia and in the 4‐(methylnitrosamino)‐1‐(3‐pyridyl)‐1‐butanone (NNK)‐induced adenoma. These findings were observed regardless of left pulmonary ligation.

Due to bleeding from the operation, a total of 32 mice in group 1 were dead before coming out from the anesthetic. Though 32 mice were killed at the termination of the experiment, a total of 21 mice with completely collapsed left lungs were counted to determine the effective number with left ligation in group 1. The effective numbers of mice in 1, 3, and 7 days were 8, 5 and 8 from groups 1 and 7, 6 and 7 mice from group 2, respectively. The weight of the right lung in mice with ligated left lungs in group 1 increased with time, and neared that of the corresponding lung in group 2 on day 7 (Figure 4). Microarray analysis of the right lungs on day seven showed that 4334 markers showed significant differences between groups 1 and 2. Functional annotation clustering revealed that there were 92 upregulated clusters in group 1, and the top 3 clusters were related to the cell cycle, cell division and mitosis (Table 2). The representative markers that showed significant changes in the right lungs in group 1 were cell division cycle 25C (Cdc25c), cytoskeleton‐associated protein 2 (Ckap2), microtubule‐associated serine/threonine kinase‐like (Mastl), and others. IGF‐1, with a fold change of 2.49 times in our experiment, is reported to play a role in cell proliferation^20^ though IGF‐1 was unclassified in the top 3 clusters of the cell cycle, cell division and mitosis. Therefore, IGF‐1, an important focus of immunohistochemical analysis, was examined in the lungs during experiment 1. A strong immunohistochemical expression of IGF‐1 was observed in the lesions of both lung hyperplasia and NNK‐induced adenoma (Figure 5). Irrespective of whether ligation occurred, the expression of IGF‐1 was observed. In the liver, the expression of IGF‐1 was observed using whole tissues, with no between‐group differences (Figure not shown).

**Table 2:** Top 3 clusters (of 92) showing the upregulation and the 10 highest expressed markers in each cluster (experiment 2)

Clusters	Function	No. of genes	*P*‐value*	Top 10 genes	Average ratio**
1	Cell cycle	98	1.15E‐57	cell division cycle 25C (Cdc25c)	2.665
				kell blood group (Kel)	2.637
				microtubule‐associated serine/threonine kinase‐like (Mastl)	2.438
				cytoskeleton‐associated protein 2 (Ckap2)	2.337
				asp (abnormal spindle)‐like, microcephaly associated (Drosophila) (Aspm)	2.277
				kinetochore associated 1 (Kntc1)	2.147
				establishment of sister chromatid cohesion N‐acetyltransferase 2 (Esco2)	2.142
				protein regulator of cytokinesis 1 (Prc1)	2.081
				non‐SMC condensin I complex, subunit G (Ncapg)	2.036
				shugoshin‐like 1 (S. pombe)(Sgol1)	1.992
				cell division cycle 25C (Cdc25c)	2.665
2	Cell division	69	5.99E‐45	microtubule‐associated serine/threonine kinase‐like (Mastl)	2.438
				asp (abnormal spindle)‐like, microcephaly associated (Drosophila) (Aspm)	2.277
				kinetochore associated 1 (Kntc1)	2.147
				protein regulator of cytokinesis 1 (Prc1)	2.081
				non‐SMC condensin II complex, subunit G2 (Ncapg2)	2.036
				shugoshin‐like 1 (S. pombe) (Sgol1)	1.992
				NUF2, NDC80 kinetochore complex component (Nuf2)	1.971
				centromere protein E (Cenpe)	1.95
				baculoviral IAP repeat‐containing 5 (Birc5)	1.877
				cell division cycle 25C (Cdc25c)	2.665
3	Mitosis	58	2.13E‐42	microtubule‐associated serine/threonine kinase‐like (Mastl)	2.438
				asp (abnormal spindle)‐like, microcephaly associated (Drosophila) (Aspm)	2.277
				kinetochore associated 1 (Kntc1)	2.147
				non‐SMC condensin II complex, subunit G2 (Ncapg2)	2.036
				shugoshin‐like 1 (S. pombe) (Sgol1)	1.992
				NUF2, NDC80 kinetochore complex component (Nuf2)	1.971
				centromere protein E (Cenpe)	1.95
				baculoviral IAP repeat‐containing 5 (Birc5)	1.877
				cyclin B1 (Ccnb1)	1.864
				cyclin B1 (Ccnb1)	1.864

* *P* < 0.05 (5.0E‐2) was considered to be significant.

## DISCUSSION

Partial pneumonectomy is reported to induce rapid and diffuse compensatory hyperplasia in the remaining parenchymal cells of the lungs to restore normal total mass, structure and function.^21^, ^22^ The mechanisms underlying compensatory hyperplasia are thought to be elevated blood flow to the remaining lobes, increased inflation of the remaining lobes, and release of soluble growth factors.^21^, ^22^ The weight of the right lungs of mice that underwent partial pneumonectomy is reported to be increased markedly a day after surgery and continued to increase steadily until postsurgical day 10, after which it was not significantly different from the total lung weight of mice that underwent sham surgery.^21^ In experiment 2, the weight of the right lung of mice with ligated left lungs in group 1 increased with time and neared the weight of the corresponding lung of mice in group 2 on day 7. These data demonstrate that the rapid and complete compensatory lung growth by left ligation in A/J mice is similar to that in left lobe pneumonectomy. In our experiments, left ligation performed by clipping the pulmonary hilum, and not left lobe pneumonectomy, was used to cause compensatory right lung growth. This clipping method was technically easy and needed less operation time. Therefore, this method seems suitably useful in bioassay experiments involving many animals. However, in our experiments, many mice were excluded owing to incomplete collapse of the left lung. Although the procedure is simple, the operator should be highly skilled and trained.

In experiment 1, hyperplasia in mice with ligated left lungs in group 1 was significantly higher than that in group 2, confirming the tumor‐promoting effect of proliferative lesions following left pulmonary ligation. The tumor‐promoting effect in lung adenoma was not confirmed, which could have been due to the low number of adenomas induced during the short period of experiment 1. Though in the present experiment, promotive effect has been confirmed only for hyperplasia but not for adenoma, our results suggest that the compensatory reaction affected potential tumor promotion and the possibility of lung tumor recurrence in the residual lung after surgical resection of the main lung tumor in humans must be examined. Generally, hyperplasia is defined as an increase in the number of cells in an organ or tissue, usually resulting in increased volume of the organ or tissue.^23^ There are two types of bronchiolo‐alveolar hyperplasia (hyperplasia) in rodent lungs.^24^, ^25^ The first is ‘inflammatory hyperplasia’ that retains its ability to revert to normal epithelia upon removal of the stimulating insult, and the second is ‘latent tumorigenic hyperplasia’, which is irreversible and causes independent preneoplastic lesions that can progress to bronchiolo‐alveolar adenocarcinoma.^24^, ^25^ This irreversible latent tumorigenic hyperplasia is recognized to be induced by NNK.^24^

In this experiment, on the other hand, the proliferative lesion‐promoting effects were not strong enough to shorten the experimental period as a lung bioassay model. If the strong promoting effect could be found in the present experiment, sequential study with left pulmonary ligation was scheduled as a next step for shortening the bioassay model. A study is also desired that employs other carcinogens or animal species. In our preliminary study, the F344 rats were examined with left pulmonary ligation after initiation with 0.1% N‐bis (2‐hydroxypropyl) nitrosamine (DHPN) in drinking water for 2 weeks. The results showed only weak promotion effect to the lung proliferative lesions and it was not enough to shorten the experimental period of bioassay model. The promotion effect by left pulmonary ligation is clearly weaker than by 2/3 partial hepatectomy of the medium‐term bioassay of the liver in rats.^11^ The liver after 2/3 partial hepatectomy in rats grows rapidly and retuned almost to the original volume after 6 weeks. However, in the left pulmonary ligation model, the volume for loss of function is less than half of total lungs and the compensatory period ends in only 2 weeks. This difference of compensatory proliferative volume and period seems to be the reason that the promotion effect by pulmonary ligation is weaker than by 2/3 partial hepatectomy.

To investigate the role of mechanical signals in the induction of gene expression after ligation, the expression of mRNA in the right lung was also examined by microarray analysis on day 7 after left pulmonary ligation in experiment 2. Day 7 was chosen for the examination because the strongest compensatory proliferation of the residual lung occurs on that day. As a result, an increasing curve was observed from the results of this experiment like that of the previous report.^21^ In our experiment, the clusters related to the cell cycle, cell division and mitosis were upregulated in group 1, and were suspected to contribute to tumor promotion. Regarding markers showing significant changes in experiment 2, IGF‐1 was focused on because it plays a role in cell proliferation.^20^ Growth hormones (GH) produced by the liver primarily stimulate the production of IGF‐1, which is the major source of circulating IGF‐1, IGFBPs, and plausibly IGF‐2 and GH. The IGF system plays a central role in the regulation of body growth and metabolic processes.^20^, ^26^, ^27^ The anti‐apoptotic and tumorigenic effect of IGF‐1 is mediated by its binding to its cognate receptor, IGF‐1R.^28^ In the experiment 2, IGF‐1 was induced as a compensatory reaction following left pulmonary ligation; the induced IGF‐1 promoted the formation of lung proliferative lesions. In experiment 1, immunohistochemical analysis of IGF‐1 in the lungs revealed strong IGF‐1 expression in the lesions, both in lung hyperplasia and the NNK‐induced adenoma, although no differences between the ligation and sham operation groups were observed. In experiment 2, the right lung examined on day 7 is supposed to be the peak of the compensatory period from the result of right lung weight, and, in experiment 1, the right lung examined in week 12 is after the compensatory period of 2 weeks after operation. This is considered to be the reason for the unclear inter‐group difference of IGF‐1 expressions for immunohistochemistry in experiment 1 and for microarray analysis in experiment 2. At least, these results suggest that IGF‐1 could be involved in the formation of lung proliferative lesions.

In conclusion, our experiments demonstrated the clear proliferative lesion‐promoting effects of pulmonary ligation, with the induction of mRNAs related to the cell cycle, cell division and mitosis; however, the proliferative lesion‐promoting effects were not strong enough to shorten the experimental period in the lung bioassay model. The results also indicate the necessity to pay attention to the recurrence of lung cancer in residual lungs after surgical resection of tumors in humans.

## DISCLOSURE STATEMENT

None declared.

## AUTHOR CONTRIBUTIONS

MY, NH, KY and YNN carried out the studies, participated in collecting data and performed the statistical analysis. MY, YM and KI participated in its design and drafted the manuscript.
